# Extreme sports performance for more than a week with severely fractured sleep

**DOI:** 10.1007/s11325-020-02172-4

**Published:** 2020-09-10

**Authors:** Nikolaus C. Netzer, Linda K. Rausch, Hannes Gatterer, Martin Burtscher, Arn H. Eliasson, Stephan Pramsohler

**Affiliations:** 1 Hermann Buhl Institute for Hypoxia and Sleep Medicine Research, University Innsbruck, Austria, Bad Aibling, Germany; 2 The Institute of Sport Science, University Innsbruck, Innsbruck, Austria; 3 Institute of Mountain Emergency Medicine, Eurac Research, Bozen, Italy; 4 Department of Medicine, Division of Sport Medicine and Rehabilitation, University Hospitals, Ulm, Germany; 5 Department of Medicine, Uniformed Services University of the Health Sciences, Bethesda, MD USA

**Keywords:** Skiing, Bicycling, Extreme sleep fragmentation, Sleep diary, Polysomnography

## Abstract

**Purpose:**

Severely fractured sleep is mostly portrayed negatively, but investigations in extreme sports show that humans can maintain performance with a minimum of sleep. With two cases of long-lasting extreme sports performances, we demonstrate that severely fragmented sleep does not necessarily lead to a deterioration of physical and cognitive performance.

**Methods:**

We performed continuous polysomnography on a 34 year-old skier for 11 days and nights during a world record attempt in long-term downhill skiing and monitored a 32 year-old cyclist during the Race Across America for 8.5 days via sleep and activity logs.

**Results:**

The skier slept fractured fashion in 15–16 naps with a daily average of 6 h consisting of 77% in sleep stage 1 and 2, 11% in stage 3, and 13% in stage REM. The cyclist slept a total of 7 h and 52 min in 8.5 days, split up into 11 short naps and 6 sleep periods. The average duration of napping was 8.8 min and of sleep 64.2 min.

**Conclusions:**

These two cases demonstrate that outstanding performances are possible with severely fractured sleep and/or sleep deprivation. In well-trained athletes, breaking new recordsis possible despite extreme sleep habits.

## Introduction

Under extreme conditions, humans are capable of performing on a high physical and mental level despite disrupted sleep or sleep deprivation, namely, in professional extreme sports events [1–5]. This observation has recently gained popular attention due to articles in the National Geographic Magazine [6]. However, it has been well established that continuous sleep fragmentation serves as a stressor for athletes in training [7, 8], and it can consequently lead to poor athletic performance as well as to mental health symptoms or disorders [9]. Although many elite athletes are frequently traveling through different time zones, a dysregulation of the circadian rhythm in the long-term is dangerous, because it can impair not only performance but also metabolism and psychological functions [9].

However, there is limited published information about the effect of disrupted sleep or sleep fragmentation on endurance racing performances of extreme sports athletes [10]. One of the few studies collecting polysomnographic data on voluntary sleep fragmentation during extreme athletic performance was conducted by Edinger et al. (2010), who monitored the sleep of two individuals during a continuous tennis match lasting for 146 h. Although the two athletes suffered from severe sleep restriction, they continued to obtain their usual amounts of slow wave sleep (SWS) throughout the marathon match [11]. Furthermore, Leger et al. (2008) assessed polysomnography (PSG) data of sailors competing in an 18-day continuous yacht race. The data showed that sleepiness during the race depended upon depth of sleep before the race [12].

The purpose of this article is to provide further information about the effect of sleep fragmentation during endurance racing performance of extreme sports athletes. 

## Methods

### Marathon downhill skier

We performed continuous PSG by the 2007 protocol of the American Academy of Sleep Medicine (AASM), manually scored by a single individual without respiratory channels in a healthy 34 year-old man for 11 days and nights during continuous downhill skiing. Sensors for leg EMG, oro-nasal airflow, and respiratory belts were not utilized, due todiscomfort of the athlete. The focus was set on the assessment of total sleep time (TST) and sleep stages; therefore EEG, EMG, and EOG data were collected. Sleep stages were defined as follows: Awake = constant ß and α rhythm (8–30 Hz, amplitude 6–120 μV) in the EEG, stage 1 and 2 = θ rhythm (20–100 μV, k-complex in 2), stage 3 = δ rhythm (0.5–3 Hz, 5–250 μV, > 20% δ), and stage REM = mainly θ rhythm (rapid eye movement in the EOG). The skiing activity contained cycles of 10 to 20 min of fast downhill skiing (speed > 50 km/h) on the slopes of Obergurgl in the Tyrolian Alps with 15–20 min lift rides giving an opportunity for napping and eating. In addition, every 48 h there was a 2-h break for eating and massage. A team of physicians, physiotherapists, sleep technicians, and other trained skiers maintained continuous monitoring of the subject.

### RAAM Marathon cyclist

We monitored a 32 year-old man during the "Race Across America" (RAAM) using sleep/activity logs. RAAM participants perform the distance of 4828 km including a climb over 53,340 m in one single stage over approximately 9 days. The athlete took sleep periods of approximately 60 min and naps of approximately 10 min whenever necessary. The exact time point of sleep periods/naps was decided during the race depending on the athlete’s physical and emotional condition, state of exhaustion, weather, and course of the road. Both athletes were interviewed by an experienced sleep physician regarding their subjective sleep quality and the length of time required to return to normal cycles of daytime wakefulness and nighttime sleep 2 weeks after the extreme sports performances. Both athletes gave written consent (including publication of pictures), and the protocol was approved by the University of Innsbruck review board. Both athletes were examined by a physician and did not show any irregularities in health parameters.

## Results

### Severely fractured sleep in marathon downhill skier

**Fig. 1:**
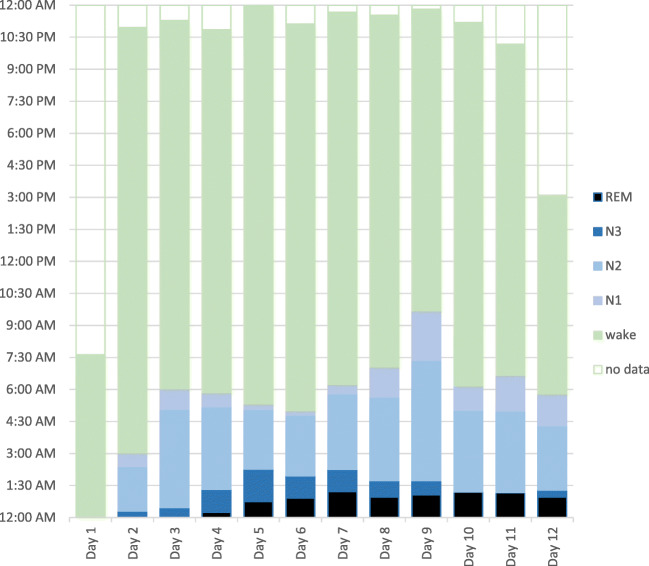
Progression of Sleep Stage Distribution in Marathon Downhill Skiing over the Duration of Twelve Days. Legend: *y*-axis shows the time of day; *x*-axis shows the distribution of sleep stages during each day; *REM* rapid eye movement sleep; *N1–N3* = non-REM sleep; *N1* = relaxed wakefulness; *N2* = light sleep; *N3* = deep sleep, slow wave sleep; classification according to the American Academy of Sleep Medicine (AASM)

PSG data collection was possible for 97% of the record attempt. The first sleep interval occurred after 10 h of skiing. During the recording time, a total sleep time of 68 h and 43 min was scored. This corresponds to an average sleep time of 6 h and 6 min per 24 h. These average sleep hours were distributed over 55 min in sleep stage N1 (17%), 3 h and 19 min in stage N2 (60%), 36 min of slow wave sleep (SWS, stage N3) (11%), as well as 42 min in stage rapid-eye-movement (REM) sleep (13%). The structure of sleep changed over time with increasing amounts of SWS, N3, and REM sleep during the first 5 days and then stabilizing. Distribution of sleep stages on different days is presented in Fig. 1. We observed the maintenance of a regular sleep stage distribution during the gondola rides and during the 2-h massage breaks. The skier accumulated approximately 15 to 16 sleep cycles per 24 h. There was only one fall by the skier during the entire event.

### Severely fractured sleep in RAAM marathon cyclist

**Fig. 2:**
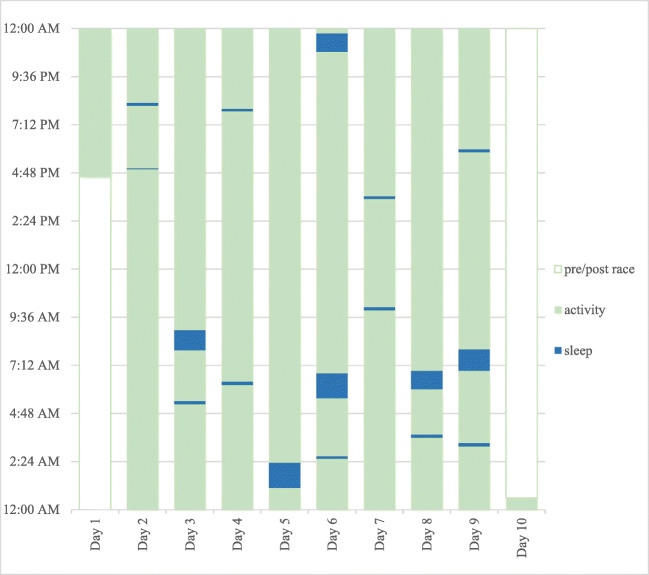
Distribution of total sleep throughout the course of the Race Across America. Legend: *y*-axis shows the passages of time during 1 day; *x*-axis shows each day of the race; pre-/post-race defines the beginning of the race at 04:18 PM (GTM-7) and the finish point at 00:35 AM (GMT-4)

Throughout the race, a total amount of 7 h and 52 min of sleep and napping was logged. The distribution of sleep during the race is presented in Fig. 2*.* Average duration of the eleven naps was 8.8 min. Average duration of the six sleep periods was 64.2 min. The athlete did not record any severe health issues or injuries throughout the RAAM.

**Fig. 3:**
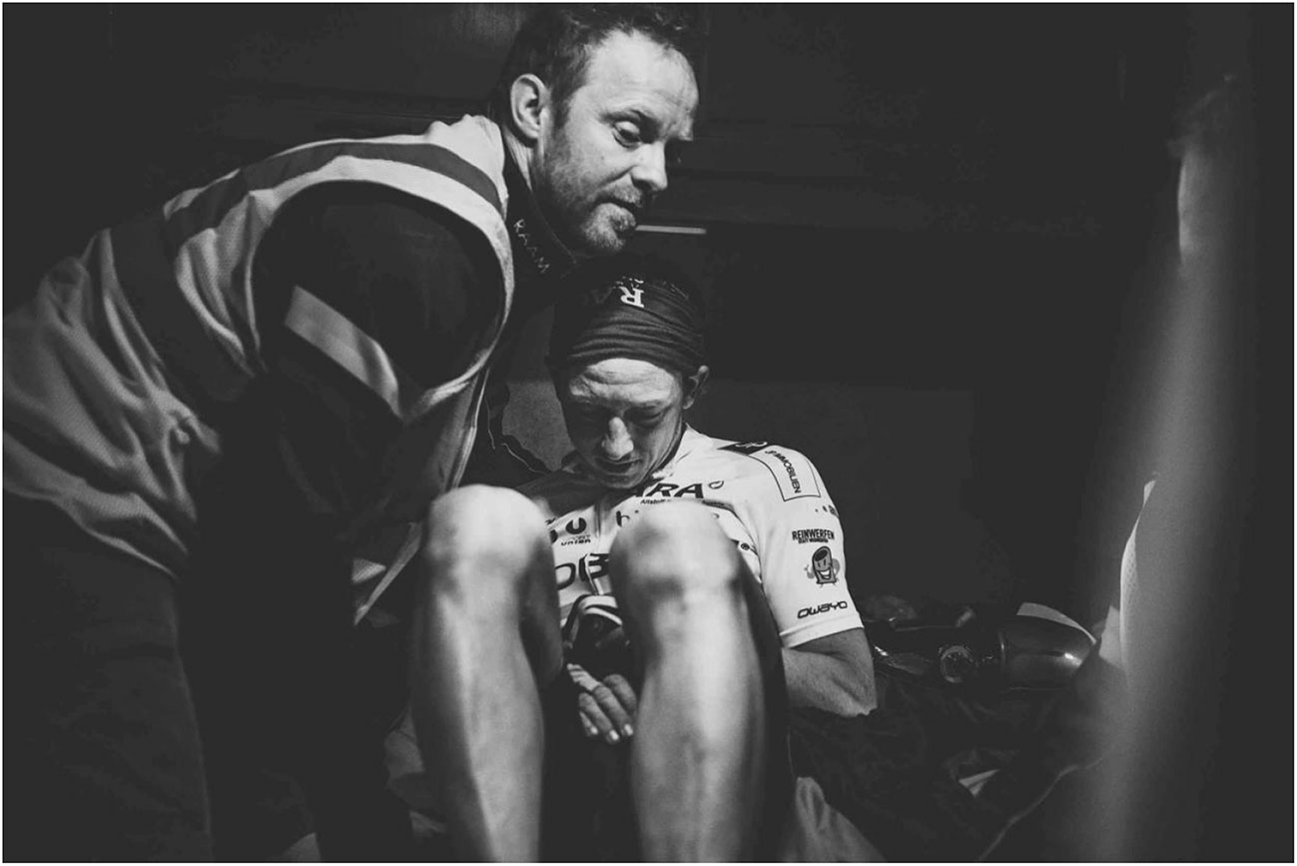
The RAAM (Race Across America) 2015 winner’s team member helping the athlete out of the bicycle saddle and into a comfortable napping position

Both athletes subjectively estimated their total sleep time for the first night after their events to be less than 3 h. They estimated their sleep onset latency for the first night to be around 1 h. They also reported frequent awakenings in the first two nights after the event. Both reported a 1-week recovery time to return to their regular circadian rhythms (Fig. 3).

## Discussion

Compared with previous PSG studies in extreme sports athletes, the monitoring of the downhill skier is the longest continuous PSG documentation of the rhythm of recurring napping in any athlete [11, 12]. Apparently, the human body is able to adapt to the rhythm of recurring napping structure over several days [13]. In the case of the marathon downhill skier, his sleeping habit adapted to the predefined 20 min resting periods during gondola rides with a healthy proportion of sleep phases resulting in reestablishing restorative functions. As an example of such sleep structure adaption in a non-sporting event, an indigenous population in South Africa, the Hazda tribe, demonstrates a similar ability to adapt sleep behavior during long distance hunts [3, 6].

Concerning the cyclist, this report is the first description of such a short total sleep time over a period greater than 8 days of extremely strenuous peak performance. In previous case studies, including another RAAM sleep report, total sleep times were longer by a multiple and with a different napping structure [1, 14]. Lahart et al. (2013) report severe emotional impairment of other RAAM athletes during the event. Although the cyclist did not report such severe impairments, the long-term health effects of sleep deprivation during such extreme sports performances are not known and could be a risk for physical and mental well-being. The unique ability of the cyclist to withstand severe sleep restriction could be of further research interest; therefore it would be necessary to obtain PSG data in order to add information to the sleep/activity logs.

As a limitation we acknowledge collection of PSG data in only one athlete. However, applying electrodes on the body of a cyclist who is performing such extreme exercise over such a long time period is not easily implemented. In comparison, we regard the application of PSG electrodes in the marathon skier as more feasible, because he had predefined resting periods due to gondola rides. A further limitation is that we do not have PSG or sleep-log data of normal nights outside of the extreme performance period. However, we expect the regular sleeping patterns in these extreme sports athletes to be ordinarily restorative; otherwise they would not be able to perform at such level.

These two cases demonstrate that outstanding performances, i.e., breaking a continuous ski record and winning the RAAM even while requiring high levels of concentration, are possible with severely fractured sleep and avoiding sleep phases much longer than 1 hour. Our observations contrast with previously described decriments in neuromuscular, hormonal, and cognitive functions after extreme performances of different types due to sleep deprivation and short continuous sleep periods [7, 8]. However, according to one of these prior publications, longer extreme sports events are associated with less neuromuscular fatigue than shorter events [8]. The latter finding may explain why the athletes described in this report could ski and cycle with good muscular control for more than a week.

## Conclusion

We conclude from both cases of professional athletes that severely fragmented sleep in healthy well-trained athletes does not necessarily lead to a significant reduction of performance of a demanding but well-trained physical task. PSG recording demonstrated that if severely fractured sleep allows for a sufficient amount of sleep, the sleep stage distribution over each 24-h period reaches levels which are commonly seen in usual day-night sleep wake cycles.

However, possible health risks appearing from sleep deprivation, such as metabolism imbalances, mental disorders, or a negative long-term effect on performance cannot be disregarded. We suggest that extreme sports athletes should take care of a healthy sleep hygiene with the help of professionals in order to cope with possible negative effects.
